# Evaluation of the Role of Human DNAJAs in the Response to Cytotoxic Chemotherapeutic Agents in a Yeast Model System

**DOI:** 10.1155/2020/9097638

**Published:** 2020-02-13

**Authors:** Aurellia Whitmore, Devon Freeny, Samantha J. Sojourner, Jana S. Miles, Willie M. Graham, Hernan Flores-Rozas

**Affiliations:** College of Pharmacy and Pharmaceutical Sciences, Florida A&M University, Tallahassee, FL, USA

## Abstract

Heat-shock proteins (HSPs) play a crucial role in maintaining protein stability for cell survival during stress-induced insults. Overexpression of HSPs in cancer cells results in antiapoptotic activity contributing to cancer cell survival and restricting the efficacy of cytotoxic chemotherapy, which continues to play an important role in the treatment of many cancers, including triple-negative breast cancer (TNBC). First-line therapy for TNBC includes anthracycline antibiotics, which are associated with serious dose-dependent side effects and the development of resistance. We previously identified *YDJ1*, which encodes a heat-shock protein 40 (HSP40), as an important factor in the cellular response to anthracyclines in yeast, with mutants displaying over 100-fold increased sensitivity to doxorubicin. In humans, the DNAJA HSP40s are homologues of *YDJ1*. To determine the role of DNAJAs in the cellular response to cytotoxic drugs, we investigated their ability to rescue *ydj1*Δ mutants from exposure to chemotherapeutic agents. Our results indicate that DNAJA1 and DNAJA2 provide effective protection, while DNAJA3 and DNAJA4 did not. The level of complementation was also dependent on the agent used, with DNAJA1 and DNAJA2 rescuing the *ydj1*Δ strain from doxorubicin, cisplatin, and heat shock. DNAJA3 and DNAJA4 did not rescue the *ydj1*Δ *strain* and interfered with the cellular response to stress when expressed in wild type background. DNAJA1 and DNAJA2 protect the cell from proteotoxic damage caused by reactive oxygen species (ROS) and are not required for repair of DNA double-strand breaks. These data indicate that the DNAJAs play a role in the protection of cells from ROS-induced cytotoxic stress.

## 1. Introduction

Despite advances in targeted therapy of cancer, cytotoxic chemotherapy remains an essential therapeutic alternative. Targeted and cytotoxic chemotherapy are two distinctive modes of cancer treatment with each associating to certain benefits and limitations. Targeted therapies are used to kill tumor cells based on the presence of cancer-specific molecules, whereas cytotoxic chemotherapy has a nonselective mechanism of action aimed at proliferating cells. However, both approaches may result in therapeutic resistance.

Some cancers lack therapeutic targets or lose them during cancer progression and therefore rely solely on cytotoxic chemotherapy as a means of treatment. This approach is used for triple-negative breast cancer (TNBC), which lacks the estrogen, progesterone, and HER2 receptors required for targeted therapy [1], limiting its treatment to the use of cytotoxic chemotherapy such as anthracycline antibiotics [2]. Anthracycline antibiotics, specifically doxorubicin, are one of the most common and effective antineoplastic agents used in treatment of a large number of malignancies.

The effectiveness of doxorubicin can be attributed to its multiple mechanisms of actions. Doxorubicin poisons DNA topoisomerase II, resulting in DNA double-strand breaks (DSBs) leading to cell death [3]. In addition, in the cell, doxorubicin is oxidized to a semiquinone, an unstable metabolite, which is recycled in a process that releases reactive oxygen species (ROS) [3]. ROS can result in a variety of effects such as lipid peroxidation, membrane damage, and DNA damage. Anthracycline-induced ROS can result in the development of cardiotoxicity, which can be partially managed by chelation of intracellular iron [3]. Doxorubicin-induced ROS trigger apoptotic pathways in nondividing cells contributing to its side effects [4]. Although effective, drug resistance to anthracyclines can develop during treatment. This resistance cannot be overcome by increasing the dose, due to potential development of cardiotoxicity [4].

Attempts to maintain efficacy while reducing toxicity of anthracyclines has been a major focus of research [5]. We have previously identified YDJ1, a homologue of the DNAJA family of Hsp40s, as a crucial factor for the protection of cells under cytotoxic stress displaying hypersensitivity (100–1000x) to protein folding from doxorubicin [6]. YDJ1 is the yeast *S. cerevisiae* HSP40 and functions as a cochaperone to HSP70. HSP40 and HSP70 together protect thermally damaged proteins from aggregation, dissociating aggregated protein complexes, refolding damaged proteins in an ATP-dependent manner, or targeting them for efficient degradation [7].

**Figure 1:**
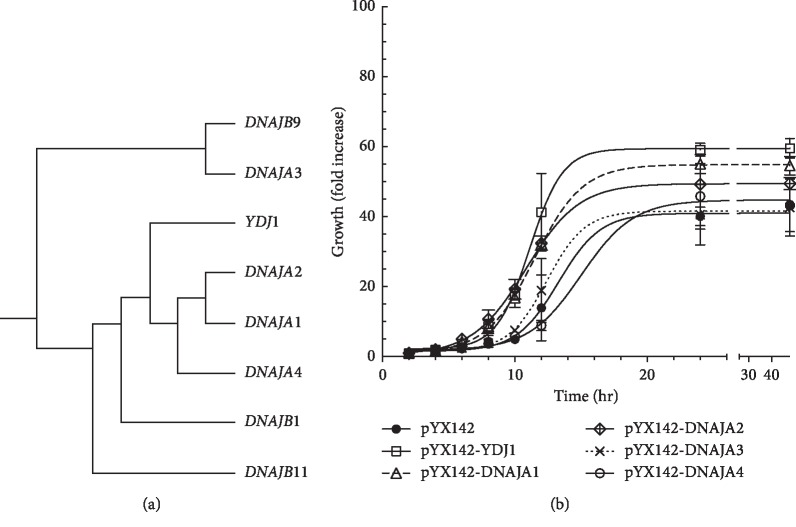
Rescue of the growth phenotype of *ydj1Δ* by the human DNAJAs. (a). Sequence comparison indicates that YDJ1 is more closely related to DNAJA1 and DNAJA 2, as indicated by the phylogenetic tree. The comparison included 49 human HSP40 sequences obtained from NCBI protein database. Comparison was performed using the Constraint-based Multiple Alignment Tool from NCBI (COBALT). (b). The growth of *ydj1*Δ complemented strains was evaluated by growing them in the absence of stressors, as described in the Materials and Methods section. The strains tested are as follows: *YDJ1* (open squares), *ydj1*Δ (closed circles), *ydj1*Δ*-*DNAJA1 (open triangles), *ydj1*Δ*-*DNAJA2 (open rhombus), *ydj1*Δ*-*DNAJA3 (--X--), and *ydj1*Δ*-*DNAJA4 (open circles). Growth was monitored at specified intervals by measuring and aliquot of the culture at OD_600_. The fold increase, relative to the initial OD_600_ of the culture, is presented.

There are 3 types of DNAJ proteins, classified based on the presence of the DNAJ domain, a zinc finger motif, a glycine/phenylalanine rich region, and a C-terminal domain. YDJ1 is most closely related to the type I subfamily DNAJA, which contains all domains/motifs [8]. Type II (DNAJB) lacks the zinc finger motif, while type III (DNAJC) only contains the J domain. There are four DNAJAs in humans, DNAJA1, DNAJA2, DNAJA3, and DNAJA4. Sequence analysis by constraint-based multiple alignment tool (NCBI, COBALT) indicates that the yeast YDJ1 is most closely related to DNAJA1 and DNAJA2 (Figure 1(a)). Pairwise analysis using the NCBI blastp suite indicates that YDJ1 is 46.23%, 46.12%, 30.95%, and 43.21% identical to DNAJA1, DNAJA2, DNAJA3, and DNAJA4, respectively.

Although a number of reports indicate that the heat-shock response prevents cytotoxic effects of doxorubicin, these have mostly focused on Hsp70 and Hsp27 [9, 10]. The role of the DNAJAs in the response to cytotoxic chemotherapy has not been investigated. Interestingly, HSP40s, including the DNAJAs, are overexpressed in multiple cancers, and recently, a report indicates that the DNAJAs have high levels of expression in breast cancer after treatment [11]. Recent work from our laboratory indicates that YDJ1, a type I HSP40 in yeast, plays a critical role in the protection from ROS stress from anthracycline exposure [12].

To determine the role of DNAJAs in the cellular response to cytotoxic drugs, we investigated their ability to rescue *ydj1*Δ mutants from exposure to chemotherapeutic agents. Mutant strains complemented by the DNAJAs were exposed to chemotherapy agents: doxorubicin, cisplatin, and etoposide, as well as oxidative stress agent menadione. Our results indicate that the different DNAJAs provide distinct levels of protection, with DNAJA1 and DNAJA2 being more effective at complementation while DNAJA3 and DNAJA4 did not complement. Deletion *ydj1*Δ strains expressing DNAJA1 or DNAJA2 survived to exposure to doxorubicin, cisplatin, and heat shock, comparable to the strain expressing the wild type YDJ1. DNAJA1 and DNAJA2 also rescued the growth phenotype of the *ydj1*Δ strain and were essential in the protection of the *ydj1*Δ strain to reactive oxygen species (ROS) generated by menadione, consistent with our previous observation in yeast [12]. Conversely, *ydj1*Δ strains harboring DNAJA3 and DNAJA4 displayed reduced survival when exposed to cytotoxic stress and did not rescue the growth phenotype of *ydj1*Δ, and in some cases, they appeared more sensitive than the noncomplemented strain. In fact, expression of DNAJA3 and DNAJA4 was detrimental to the growth of the wild type strain and sensitized it to both doxorubicin and heat shock, suggesting that DNAJA3 and DNAJA4 interfere with the normal heat-shock response in yeast. Our results indicate that DNAJA1 and DNAJA2 are functional homologues of yeast YDJ1 and play a role in the protection of cells from cytotoxic stresses such as those exerted by cancer chemotherapeutic agents.

## 2. Materials and Methods

### 2.1. Media and Chemicals

*E. coli* strains were grown in LB broth or on LB agar, both supplemented with 100 *µ*g/ml ampicillin (Sigma-Aldrich) for plasmid maintenance, when appropriate. Yeast strains were grown in leucine drop-out media (Leu^−^) for plasmid selection, containing 0.67% yeast nitrogen base, 2% agar, 2% glucose (dextrose) or 2% galactose, and 0.087% amino acid drop-out mix [13, 14]. When required, etoposide (Chem-Impex International Inc.) was included in Leu^−^ selective media at 1 mM concentration. Doxorubicin-HCl (2 mg/mL) was purchased from MP Biomedicals (Irvine, CA, USA); cisplatin (1 mg/mL) was purchased from Calbiochem; menadione (Vitamin K3) was purchased from Enzo Life Sciences (Farmingdale, NY); etoposide was purchased from Chem-Impex International Inc (Wood Dale, IL).

### 2.2. *S. cerevisiae* Strains

The genotypes of all strains used in these studies are shown in Table 1. Homozygous haploid deletion strains library (parental strain BY4741: *MATa his3*Δ*1 leu2*Δ*0 met15*Δ*0 ura3*Δ*0*) was obtained from Thermo Fisher Scientific (Waltham, MA). The gene deletion present in the strains used in this study have been validated by polymerase chain reaction (data not shown).

### 2.3. Molecular Biology

The *YDJ1* gene was PCR-amplified using genomic DNA from wild type strain BY4741 as the template and cloned into the EcoRI/SacI restriction sites of the pXY142 yeast expression vector (Ingenious, 2*μ*, LEU2, TPI constitutive expression promoter). Correct clone was confirmed by DNA sequencing (MCLAB, San Francisco, CA). The DNAJAs 1 to 4 were PCR-amplified from plasmids harboring the genes (Dharmacon, Lafayette, CO) and cloned into the NcoI/SacI restriction sites of pYX142. Correct clones were confirmed by DNA sequence.

### 2.4. Yeast Genetics and Cytotoxic Stress Sensitivity Assays

HSP40s-expressing plasmids were transformed into *ydj1*Δ or wild type strains, as previously described [1, 6, 15–19]. The *rad52*Δ deletion strain was transformed with pYX142 empty vector to provide LEU2 selectivity and served as a control for sensitivity to DNA double-strand breaks (DSBs) by etoposide.

For the growth rate analysis, cells harboring the expression plasmid were cultured overnight in selective media as described above (at 30°C, for approximately 16 hours) to saturation, and then new cultures were started by inoculating with the overnight culture to a dilution of OD_600_ = 0.04. The cultures were started (30°C, with shaking) and aliquots were taken to measure the OD_600_, at timed intervals and observed under the microscope to exclude bacterial contamination. In the cytotoxic stress survival assay, the concentration of the drugs used for strain exposure was determined experimentally using the WT parental strain BY4741 and sensitive strain *ydj1*, as previously described [6]. Single colonies were grown overnight in liquid Leu^−^ media, at 30°C with shaking. Cells were then washed and resuspended in ultrapure sterile water. Strains were then separated into control and treatment groups and exposed to drug or vehicle for 1–3 h depending on the agent. After exposure, the cells were once again washed and suspended in sterile water. Serial dilutions (20 *µ*L) were spotted onto Leu^−^ agar plates and incubated at 30°C. Heat-shock treatment was performed by plating serial dilutions of the strains and incubating at 37°C. Cell growth was monitored daily, and colonies were counted at day 3. Survival was calculated relative to the corresponding untreated control, and sensitivity was determined relative to the survival of the *ydj1*Δ*-*complemented strain (YDJ1). Survival, as indicated in the Results section, is specific for that drug concentration. Each trial involved the testing of five independent colonies for each cytotoxic agent, and a minimum of three trials were performed. The survival of the untreated strain was defined as 100%.

### 2.5. Statistical Analysis

Data analysis and graphing was performed using the GraphPad Prism 7 software package. Specific analysis for each experiment is indicated in the respective figure. The mean of at least three trials is plotted, together with the SEM. Differences between mean values and multiple groups were analyzed by one-way analysis of variance (ANOVA). Statistical significance was set at *p* < 0.05. Fitting and interpolation of the sigmoidal growth curve were performed using the model Sigmoidal, 4PL, X is log (concentration) from GraphPad Prism 7.

## 3. Results

### 3.1. Rescue of *ydj1*Δ Growth Phenotype by the Human DNAJAs

The *ydj1*Δ strain displays a growth defect, which results in slow growth, longer doubling time, and small colonies relative to the wild type. To determine if the human DNAJAs can rescue the growth defect of *ydj1*Δ, the strain was transformed with yeast expression plasmids expressing each DNAJA (1–4) as well as *YDJ1* (positive control) and empty vector (negative control) and cultured as described in Materials and Methods section. Growth showed a typical sigmoidal curve with varying lag time, slope, and plateau, depending on the strain. Several parameters, including top plateau, Growth_50_, hill slope, and span (see Table 2), were derived using the Sigmoidal 4PL model equation from GraphPad Prism 7, where *X* is the log (concentration). As shown in Figure 1, the *ydj1*Δ strain shows a long lag time, compared to the complemented strain (*YDJ1*), with slow growth rate at exponential phase (hill slope of 0.281 vs 0.347), slower half maximum growth (growth 50%), achieved in 13.2 vs 11.11 hours, respectively, and lower maximum growth (plateau) (40.2-fold increase vs 59.4-fold, respectively) (Table 2). Interestingly, DNAJA1 and DNAJA2 effectively complemented the *ydj1*Δ strain, achieving similar maximum growth (55.2-fold and 49.4-fold increase, respectively) compared to the *YDJ1* complemented strain (59.4). They achieved half maximum growth at similar times (11.5 h and 10.8 h) to the *ydj1*Δ-complemented strain (YDJ1, 11.1 h), although they showed slightly lower growth rate at the exponential phase (0.238 and 0.217, respectively). The span, which represents the difference between the top and the bottom plateau, shows that the overall performance of each strain corresponds to the maximum growth they can achieve. However, strains expressing DNAJA3 and DNAJA4 resembled more the *ydj1*Δ strain, achieving low maximum growth (38.9-fold increase for DNAJA3 and 44.0 for DNAJA4) and long half maximum growth time (12.3 h). This is more evident in the DNAJA4-complemented strain, with half maximum growth time of 15.0 h.

### 3.2. Complementation of the sensitivity of the *ydj1*Δ mutant strain to chemotherapeutic agents doxorubicin and cisplatin by human DNAJAs

**Figure 2:**
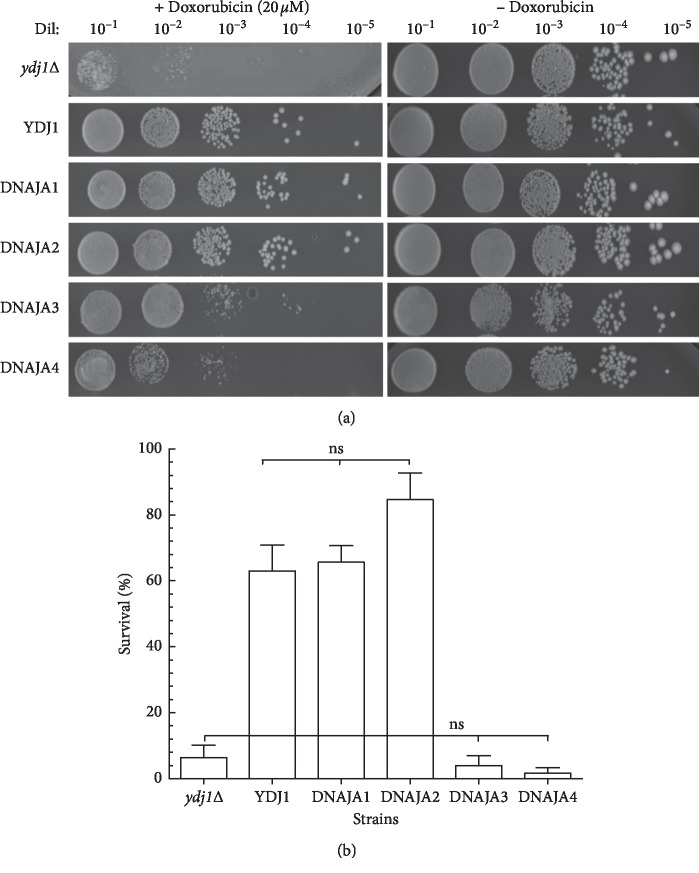
Sensitivity of the DNAJAs-expressing strains to doxorubicin. (a) The survival of the strains to 20 μM doxorubicin was determined as described in the “Materials and Methods” section. Serial dilutions (1 : 10–1 : 10^5^) of the treated cultures were spotted onto Leu^−^ + glucose plates. Growth was scored after 3 days of incubation at 30°C. The serial dilutions of the strains are shown. (b) Quantification of the survival of the tested strains. Survival was determined by counting the number of colonies in the respective dilutions and calculated on the basis of the growth of strains not treated with doxorubicin. At least three sets of experiments were used in the statistical analysis. Average survival plus standard deviation is shown. Dil: serial dilutions; Doxo: doxorubicin; ns: not significantly different.

We have previously shown yeast strains deleted in the HSP40 *YDJ1* to be highly sensitive to cytotoxic stress [6]. To evaluate the ability of DNAJAs to complement the sensitivity of *ydj1*Δ, we determined the survival of the *ydj1*Δ strains expressing each human DNAJA exposed to chemotherapeutic agents doxorubicin and cisplatin. The concentration of the drugs used in the assays was determined empirically using the wild type strain (not sensitive control) and *ydj1*Δ (sensitive control). Strains were grown in Leu- selective media to maintain the expression plasmid, treated and washed to remove the drug as described in Material and Methods section. Serial dilutions were spotted in Leu- agar plates to count colonies and to determine survival. When exposed to doxorubicin (20 *μ*M), the strain harboring a wild type copy of *YDJ1* displayed 63% survival relative to the untreated strain. As expected, the *ydj1*Δ strain was highly sensitive with 6.4% survival, which is 10-fold more sensitive than the complemented strain (YDJ1) (Figures 2(a) and 2(b) and Table 3). Interestingly, both DNAJA1 and DNAJA2 were effective at complementing the *ydj1*Δ mutant, displaying similar levels of survival to the strain expressing the wild type gene (66% and 85% survival for DNAJA1 and DNAJA2, respectively). However, the strains expressing DNAJA3 and DNAJA4 were sensitive to doxorubicin (4% and 1.7% survival, respectively), similar to the noncomplemented *ydj1*Δ strain. In fact, the strain expressing DNAJA4 appears more sensitive (37-fold higher sensitivity compared to the *YDJ1* strain) (Figures 2(a) and 2(b) and Table 3). While the survival of *YDJ1-* and DNAJA1-complemented strains is not statistically significantly different (*p* > 0.05) among each other, they are significantly different to the *ydj1*Δ strain (*p* < 0.05). Interestingly, the DNAJA2-complemented strain displays higher survival indicating that DNAJA2 provides more fitness than the wild type YDJ1 itself.

**Figure 3:**
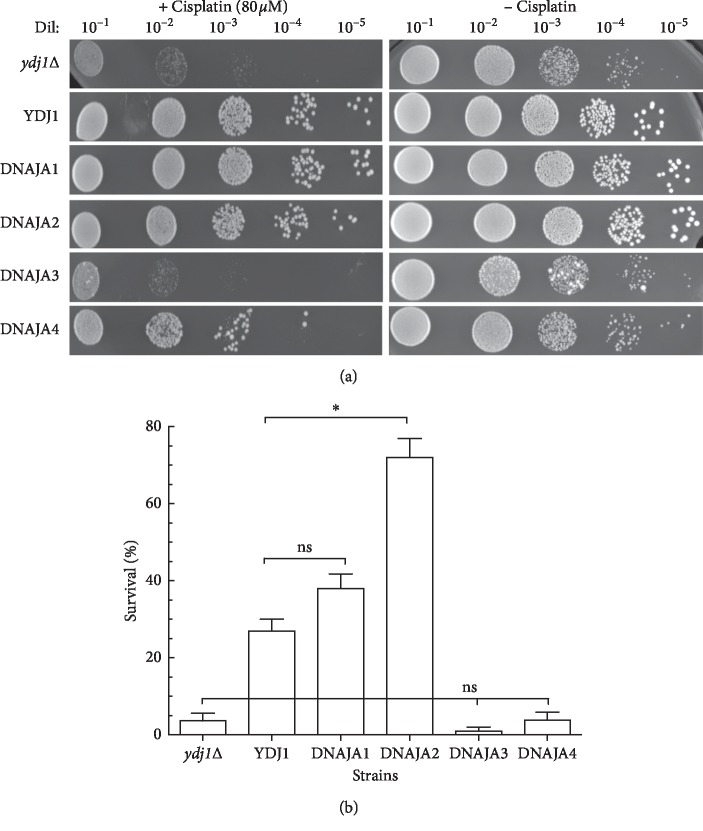
Sensitivity of the DNAJAs-expressing strains to cisplatin. (a) Strains were exposed to 80 *μ*M cisplatin. Serial dilutions (1 : 10–1 : 105) of the treated cultures were spotted onto Leu^−^ + glucose plates. Growth was scored after 3 days of incubation at 30°C. The serial dilutions of the strains are shown. (b) Quantification of the survival of the tested strains. Survival was determined by counting the number of colonies in the respective dilutions and calculated on the basis of the growth of strains not treated with cisplatin. At least three sets of experiments were used in the statistical analysis. Average survival plus standard deviation is shown. Dil: serial dilutions; Cis: cisplatin; ns: not significantly different. ^*∗*^Significantly different.

Exposure of the strains to cisplatin (80 *μ*M) indicates that both the DNAJA1- and DNAJA2-complemented strains rescued the sensitivity of the *ydj1*Δ mutation (*p* < 0.05). While DNAJA1 displayed a survival similar to that of the strain complemented by *YDJ1* (38% vs 27%, respectively), the DNAJA2-complemented strain was significantly more resistant to cisplatin (72% survival) (Figures 3(a) and 3(b) and Table 3). As with doxorubicin, the strains expressing DNAJA3 and DNAJA4 failed to complement the *ydj1*Δ mutation, with the DNAJA3 strain showing significant sensitivity (1% survival) which is 27-fold more sensitive than the *YDJ1*-complemented strain and ∼3-fold more sensitive than the *ydj1*Δ strain (Table 3). The DNAJA4-expressing strain was similar to *ydj1*Δ (3.7% vs 4% survival, respectively) (Figures 3(a) and 3(b) and Table 3).

**Figure 4:**
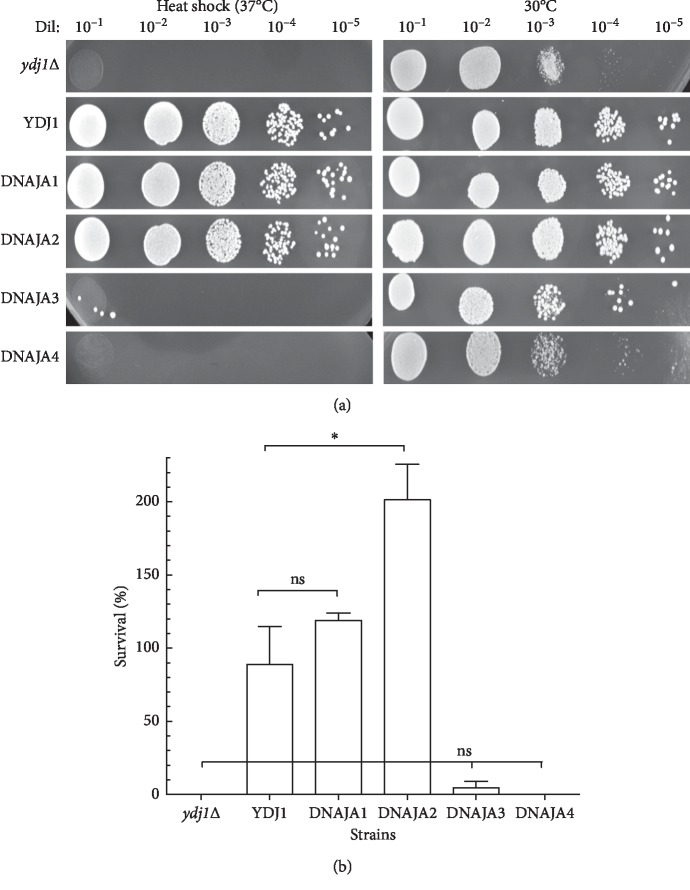
Sensitivity of the DNAJAs-expressing strains to heat shock. (a) The survival of the strains was tested for heat sensitivity. Serial dilutions of the cells were plated onto Leu^−^ + glucose plates and incubated at 30°C (untreated controls) and at 37°C (heat shock). Growth was scored at 72 hours. YDJ1 is the positive control, resistant to heat shock, and *ydj1*Δ is the negative control, sensitive to heat-shock. (b) Survival was determined by growth of the heat-shocked strain relative to the growth of nonheat-shocked cells. At least three sets of experiments were used in the statistical analysis. Average survival plus standard deviation is shown. Dil: serial dilutions; ns: not significantly different. ^*∗*^Significantly different.

The role of YDJ1 in the heat-shock response has been clearly described. The *ydj1*Δ deletion strain is highly sensitive to heat shock and does not survive exposure to 37°C (heat shock for yeast). We tested the ability of the human DNAJAs to rescue the heat-sensitive phenotype of *ydj1*Δ. As shown in Figures 4(a) and 4(b), the *ydj1*Δ strain shows no growth upon heat shock, while complementation with *YDJ1*, DNAJA1, and DNAJA2 rescues the phenotype. In fact, the DNAJA2-expressing strain grows significantly better at 37°C (Figures 4(a) and 4(b) and Table 3). Conversely, DNAJA3 and DNAJA4 failed to rescue *ydj1* from heat shock and similarly showed no growth at 37°C (Figures 4(a) and 4(b) and Table 3). These data suggest that both DNAJA1 and DNAJA2 are functional homologs of *YDJ1* and can substitute it effectively, rescuing the defects of the deletion strain.

### 3.3. Sensitivity of DNAJA1- and DNAJ2-Expressing Strains to Menadione and Etoposide

Doxorubicin exerts its antineoplastic activity through two main mechanisms: (i) DNA damage (generation of DSBs) and (ii) by the generation of reactive oxygen species (ROS) [4]. Our results indicate that both DNAJA1 and DNAJA2 were more effective at protecting the *ydj1*Δ strain from doxorubicin. To further investigate if the protection was specific to DSBs or ROS, we tested the sensitivity of the strains to the oxidative stress generating agent menadione and to the DNA-topoisomerase II inhibitor, etoposide.

**Figure 5:**
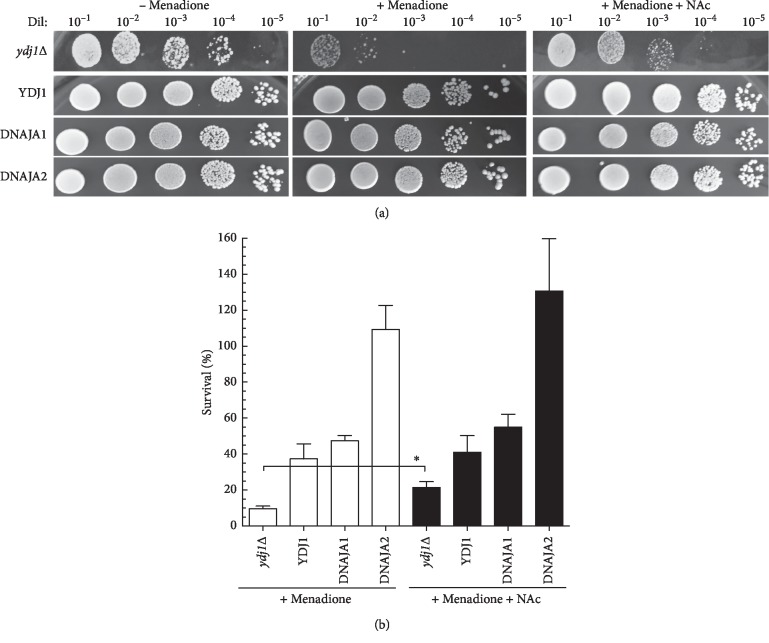
Sensitivity of the DNAJAs-expressing strains to menadione. (a) DNAJA1- and DNAJA2-expressing strains showed the most resistance to doxorubicin and cisplatin and were therefore exposed to 6.6 mM menadione (1 h at 30°C) to determine survival. Cells were washed with sterile water and serial dilutions (1 : 10–1 : 10^5^) of the treated cultures were spotted onto Leu^−^ + glucose plates. Growth was scored after 3 days of incubation at 30°C. The serial dilutions of the strains are shown. N-acetylcysteine (NAc, 20 mM) was added as cotreatment with menadione to the indicated strains. (b) Quantification of the survival of the tested strains. Survival was determined by counting the number of colonies in the respective dilutions and calculated on the basis of the growth of strains not treated with menadione. At least three sets of experiments were used in the statistical analysis. Average survival plus standard deviation is shown. Dil: serial dilutions. ^*∗*^Significantly different.

Menadione is commonly used in research as a ROS-producing agent and shares the same quinone ring as doxorubicin [4]. As shown in Figure 5(a), the strains that displayed the highest levels of protection from exposure to menadione were those expressing DNAJA2 (109% survival) and DNAJA1 (47% survival), corresponding to 0.3-fold and 0.8-fold sensitivity relative to the YDJ1 (37%), respectively. The *ydj1*Δ strain is highly sensitive to oxidative stress, displaying 10% survival (>3-fold more sensitive than YDJ1). Addition of ROS scavenger agent N-acetylcysteine (NAc) significantly increased the survival of the *ydj1*Δ strain (21%), confirming that this strain is sensitive to ROS (Table 4, Figures 5(a) and 5(b)). However, in HSP40-complemented strains (YDJ1, DNAJA1, and DNAJA2), NAc only had a marginal effect (∼20% increase in survival, Figures 5(a) and 5(b)) Our results indicate that DNAJA1 and DNAJA2 protect the cell viability from exposure to ROS-generating agents such as menadione.

**Figure 6:**
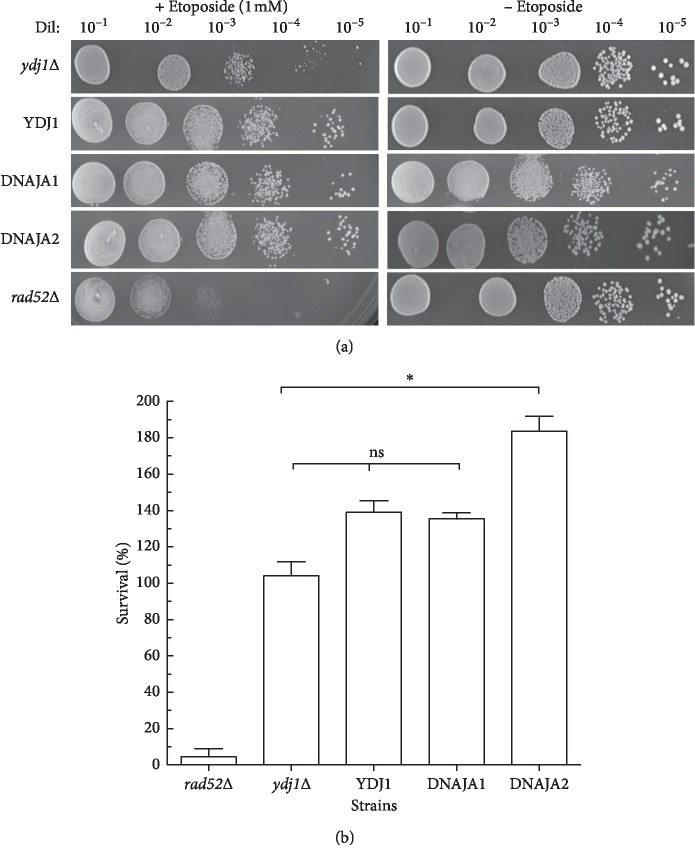
Sensitivity of the DNAJAs-expressing strains to etoposide. (a) The survival of DNAJA1 and DNAJA2 strains was determined through exposure to etoposide. Serial dilutions of the treated strains were spotted onto Leu^−^ + glucose plates containing etoposide (1 mM) and incubated at 30°C. Growth was scored at 72 hours. Positive controls sensitive to etoposide is the *rad52*Δ deletion strain. (b) Survival was determined by growth of the treated strain relative to the growth of its untreated control. At least three sets of experiments were used in the statistical analysis. Average survival plus standard deviation is shown. Dil: serial dilutions; ns: not significantly different. ^*∗*^Significantly different.

As doxorubicin, the topoisomerase II inhibitor etoposide generates DSBs that require homologous recombination for repair. As expected, the homologous recombination defective mutant *rad52*Δ is highly sensitive to the drug (5% survival) (Figures 6(a) and 6(b), Table 4). However, the *ydj1*Δ strain does not show sensitivity to etoposide (104% survival) and the expression of the wild type *YDJ1*, or DNAJA1 and DNAJA2, does not negatively affect this survival (139%, 135%, and 184%, respectively), with DNAJA1 not statistically significantly different to the strain complemented with wild type YDJ1. The survival of the DNAJA2-complemented strain was significantly higher than that of YDJ1-complemented strain (Figures 6(a) and 6(b) and Table 4).

Together, these data indicate that DNAJA1 and DNAJA2 are effective at complementing the *ydj1*Δ mutation, rescuing it from its sensitivity to doxorubicin, cisplatin, and heat shock, most likely through its chaperone activity.

### 3.4. Distant DNAJA3 and DNAJA4 Interfere with YDJ1 in Wild Type Cells

**Figure 7:**
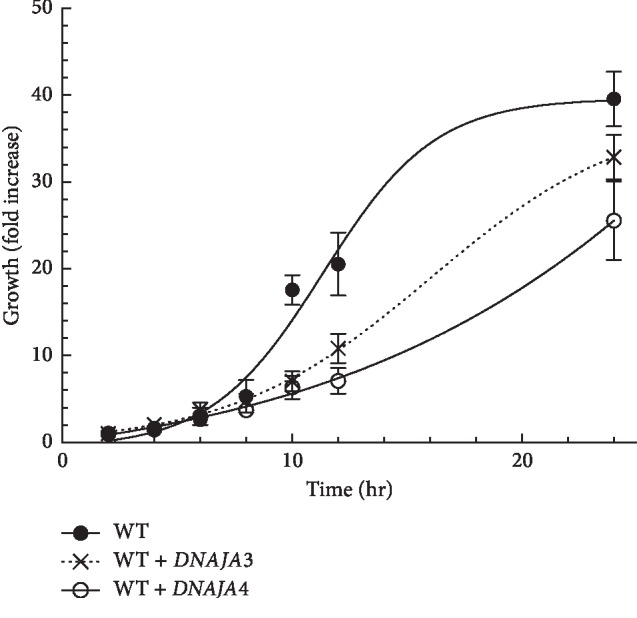
Effect of DNAJA3 and DNAJA4 in the growth of cells with wild type YDJ1. Growth of DNAJA3- and DNAJA4-expressing cells was evaluated under nonstress conditions Wild type cells contained an empty plasmid to provide selection (black circles), a plasmid expressing DNAJA3 (--X--), or a plasmid expressing DNAJA4 (open circles). Growth was monitored at specified intervals by measuring and aliquot of the culture at OD_600_. The fold increase, relative to the initial OD_600_ of the culture, is presented.

**Figure 8:**
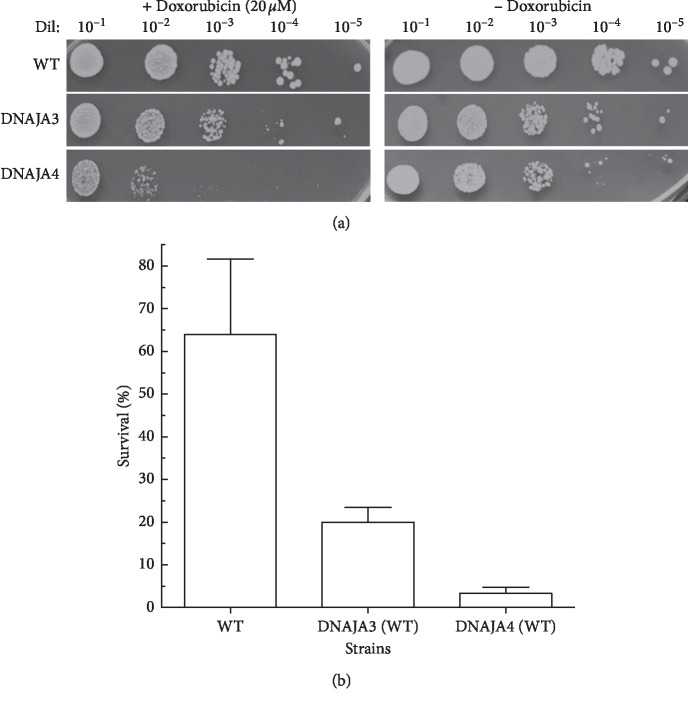
Effect of DNAJA3 and DNAJA4 expression in cells with wild type YDJ1 to exposure to doxorubicin. (a) The survival to doxorubicin exposure of the DNAJA3- and DNAJA4-expressing wild type cells was determined. Serial dilutions (1 : 10–1 : 10^5^) of the treated cultures were spotted onto Leu^−^ + glucose plates. Growth was scored after 3 days of incubation at 30°C. The serial dilutions of the strains are shown. (b) Quantification of the survival of the tested strains. Survival was determined by counting the number of colonies in the respective dilutions and calculated on the basis of the growth of strains not treated with doxorubicin. At least three sets of experiments were used in the statistical analysis. Average survival plus standard deviation is shown. Dil: serial dilutions; Doxo, doxorubicin.

Our results indicate that both DNAJA3 and DNAJA4 fail to complement the *ydj1*Δ mutant as DNAJA1 and DNAJA2 do. In fact, strains expressing DNAJA3 and DNAJA4 appear as sensitive or more sensitive than the deletion strain. Based on these results, it is possible that DNAJA3 and DNAJA4, which are more distant homologs of *YDJ1* than DNAJA1 and DNAJA2, may be interfering in *YDJ1*-dependent functions. To confirm this possibility, we proceeded to express DNAJA3 and DNAJA4 in a wild type background with functional *YDJ1*. As shown in Figure 7, expression of DNAJA3 or DNAJA4 considerably reduced the growth rate of the wild type strain, with a hill slope, at the exponential phase, of 0.18 for wild type and 0.04 and 0.10 for WT + DNAJA3 and WT + DNAJA4, respectively. The time to get to 50% growth is also extended from 11.34 h for wild type to ∼14 h and 16 h for WT + DNAJA3 and WT + DNAJA4, respectively. To evaluate if these distant DNAJAs affect the response of wild type cells to cytotoxic stressors, we determined the survival of WT + DNAJA3 and WT + DNAJA4 strains after exposure to doxorubicin and heat shock (Figures 8(a) and 8(b)). Expression of DNAJA3 or DNAJA4 significantly reduced the survival of the wild type strain from 63% to 20% for WT + DNAJA3 (3-fold more sensitive, Table 5) and 4% for WT + DNAJA4 (15-fold more sensitive, Table 5), both significantly different than the wild type (*p* < 0.05).

**Figure 9:**
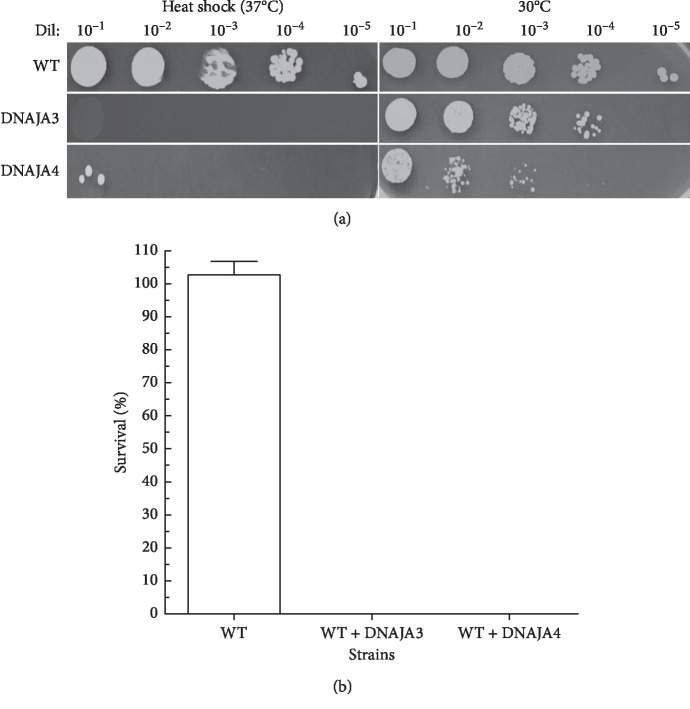
Effect of the expression of DNAJA3 and DNAJA4 in cells with wild type YDJ1 exposed to heat shock. (a) The survival to heat shock of the DNAJA3- and DNAJA4-expressing wild type cells containing was determined. Serial dilutions of the cells were plated onto Leu^−^ + glucose plates and incubated at 30°C (untreated controls) and at 37°C (heat shock). Growth was scored at 72 hours. WT is the positive control, resistant to heat shock. (b) Survival was determined by growth of the heat-shocked strain relative to the growth of nonheat-shocked cells. At least three sets of experiments were used in the statistical analysis. Average survival plus standard deviation is shown. Dil: serial dilutions; Doxo, doxorubicin.

The expression of DNAJA3 and DNAJA4 in a wild type background also interfered with the response to heat shock (Figures 9(a) and 9(b)). Both the WT + DNAJA3 and WT + DNAJA4 strains did not survive the heat shock (0% survival) compared to the wild type (104% survival). Our data confirm that the expression of the distant HSP40s, DNAJA3 and DNAJ4, interferes with a functional *YDJ1* affecting the response of the cell to stress and displaying a phenotype similar to that of the *ydj1*Δ strain.

## 4. Discussion

Cytotoxic therapeutic agents, such as doxorubicin and cisplatin, are commonly used as sole agents or in combination therapy in cancers that lack biological targets. However, their nonselective mechanism of action against cancer cells also results in serious side effects such as cardiotoxicity, nephrotoxicity, and diverse injuries to healthy tissues that can lead to necrosis [20]. Significant effort in the field of cancer therapeutics is aimed at increasing the effects of cytotoxic agents to cancer cells while mitigating their toxic effects.

One approach to the goal of increasing therapeutic efficacy of anthracyclines, while decreasing toxic effects, is through the hypersensitization of cancer cells. A study in 2001 described high levels of Hsp40 in the serum of lung cancer patients compared to the serum of patients with no lung cancer [21]. Moreover, there is increasing evidence of HSP40s overexpression in a plethora of metastatic tumors including, among others, those of the breast, prostate, brain, and lung [22]. It has been suggested that the proteins in cancer cells depend heavily on HSPs due to protein misfolding brought in by acquired mutations that result in altered protein structure. This is critical for factors necessary to support rapid proliferation and survival of cancer cells driven by oncoproteins promoting metastatic growth [1]. Understanding the role of Hsp40s is vital for targeted inhibition of their overexpression as a potential therapeutic option. Reducing the levels of overexpressed HSP40s would sensitize cancer cells to therapeutic agent and lead to a lower effective therapeutic dosage of cytotoxic drugs resulting in less toxicity and thus enhancing the patient's quality of life.

Previous work in our lab identified YDJ1, a homologue of the DNAJA-type Hsp40s, as a crucial factor for survival under doxorubicin stress. We have extended our investigation by evaluating the role of all human DNAJAs (DNAJA1, DNAJA2, DNAJA3, and DNAJA4) in the response of cells to cancer therapeutic agents, such as doxorubicin, cisplatin, and etoposide, and to define cytotoxic stresses such as ROS and heat shock.

While YDJ1 is more closely related to the DNAJA subfamily of HSP40s, than to DNAJB and DNAJC subfamilies, there are differences between the homology of YDJ1 and the DNAJA subfamily members. Phylogenetic analysis indicates that DNAJA1 and DNAJA2 are closer sequence homologues, while DNAJA3 and DNAJA4 are more distantly related. In order to determine functional homology, we tested if the DNAJAs could rescue the phenotype of the *ydj1*Δ deletion strain by expressing them in this strain and exposing them to diverse cytotoxic stresses. As described in the Results section, DNAJA1 and DNAJA2 displayed the highest levels of complementation and were found to consistently protect cells from all agents tested to levels similar to those of wild type YDJ1. The requirement for HSP40s in normal cell growth has been well documented [16]. In fact, cells lacking *YDJ1* display a slow growth phenotype, as indicated by a longer doubling time, reduced maximal growth in culture, and formation of smaller colonies on solid agar plates. Once again, DNAJA1 and DNAJA2 could rescue the growth phenotype, while DNAJA3 and DNAJA4 could not. Consistently, DNAJA3 and DNAJA4 failed to complement the *ydj1*Δ strain, and when expressed in wild type cells, they interfered with the endogenous pathway, affecting cell growth and sensitizing the cell to stress. It is possible that DNAJA3 and DNAJA4 form nonproductive interactions with components of the heat-shock response (namely, HSP70s), sequestering and preventing them from performing YDJ1 independent functions that are crucial for cell growth. In fact, there are at least 22 HSP40s [23] and multiple HSP70s [24] which do not have exclusive partners and interact with each other.

As expected, the HSP40s are not required for the repair of DSBs, since the *ydj1*Δ deletion strain was not sensitive to etoposide. However, they are essential for the survival to exposure to ROS-generating agent menadione, indicating that ROS-induced protein damage is processed by DNAJA1 and DNAJA2.

While the HSP40s have not been as extensively studied as HSP90 and the HSP70s, recent interest in these chaperones has increased our knowledge of their function and the cellular processes they are involved in, besides their protein folding roles. DNAJA1, as all DNAJA members, is induced by heat shock factor 1 (HSF1). DNAJA1 negatively regulates the translocation of BAX from the cytosol to mitochondria in response to cellular stress, thereby protecting cells against apoptosis, and has subcellular localization within the nucleus, mitochondria, and endoplasmic reticulum [25]. It is known for binding to ubiquitin protein ligase and chaperone activity [26]. There are several DNAJA1 isoforms, one of which, isoform 2, is highly expressed in the testes and lung [27].

DNAJA2 has been shown to play a role in the positive regulation of cellular proliferation and the refolding of proteins and subcellular localizations within the cytosol [28]. It catalyzes unfolded protein binding and heat shock protein binding activity [29]. DNAJA2 is highly expressed in the adrenal gland, duodenum, kidney tubules, testis seminiferous ducts, and follicle ovarian cells [30].

DNAJA3 is localized to mitochondria and mediates several cellular processes including proliferation, survival, and apoptotic signal transduction [31]. It plays a critical role in tumor suppression through interactions with oncogenic proteins including ErbB2 and the p53 tumor suppressor protein [32]. DNAJA3 has been found to bind to protein kinase [33]. Its subcellular locations include postsynaptic plasma membrane, cytosol, and mitochondria. Its expression is high within the heart, liver, lung, and skeletal muscles with expression in keratinocytes. DNAJA3 has been shown to play a crucial role in preventing dilated cardiomyopathy [34].

DNAJA4 has negative regulation of inclusion body assembly [35]1). It interacts with nonstructure 2 protein of classical swine fever virus by 2-hybrid system [36]. It has been shown to be involved in cholesterol biosynthesis as a SREBP-regulated chaperone [37]. DNAJA4, as well as DNAJA1 and DNAJA2, acts in concert with Hsc70 to regulate the maturation and trafficking of hERG potassium channels [38].

Molecular chaperones such as DNAJAs are involved in the regulation of kinases, caspases, and other protein remodeling events, and it has been proposed that altered levels of HSP expression in cancer could lead to the loss of control of cell growth and inhibitory effects on apoptosis [17]. In fact, altered expression of HSPs has been reported for almost all classes of tumors, and because of their role in the control of cell growth, they could serve as biomarkers for cancer diagnosis and therapy [17].

The role of the DNAJAs in the response to stress may be associated to the specific client proteins they interact with and the biological processes they participate in. The role of the heat shock response in preventing cytotoxicity of doxorubicin has been reported; however, these studies have mostly focused on Hsp70 and Hsp27 [9]. Our study has identified the *YDJ1* homologues DNAJA1 and DNAJA2 as crucial factors for survival from doxorubicin and cisplatin stress. Interestingly, while anthracyclines act through a combination of DNA damage and generation of ROS, preventing the cell from responding to ROS-induced protein damage is sufficient sensitize it. Additionally, increasing the expression of these DNAJAs may provide protection in noncancerous sensitive tissue. Future research will elucidate the role of these genes in mammalian cells. Targeting these factors for chemotherapeutic sensitization of cancer cells may have potential in the development of alternative therapeutic treatments.

## Figures and Tables

**Table 1:** Yeast strains used in this study.

Strain	Genotype	Description
Wild type (WT)	*MATa his3-1 leu2*Δ *met15*Δ *ura3*Δ *[pYX142 LEU2]*	Parental *S. cerevisiae* strain (BY4741)
*ydj1*Δ	*MATa his3-1 leu2*Δ *met15*Δ *ura3*Δ *ydj1*Δ *[pYX142 LEU2]*	*ydj1* deletion strain
YDJ1	*MATa his3-1 leu2*Δ *met15*Δ *ura3*Δ *ydj1*Δ *[pYX142-YDJ1 LEU2]*	*ydj1* deletion strain complemented with wild type *YDJ1* gene
*ydj1-*DNAJA1	*MATa his3-1 leu2*Δ *met15*Δ *ura3*Δ *ydj1*Δ *[pYX1423-ydj1-DNAJA1 LEU2]*	*ydj1* deletion strain complemented with human DNAJA1 gene
*ydj1-*DNAJA2	*MATa his3-1 leu2*Δ *met15*Δ *ura3*Δ *ydj1*Δ *[pYX142-ydj1-DNAJA2 LEU2]*	*ydj1* deletion strain complemented with human DNAJA2 gene
*ydj1-*DNAJA3	*MATa his3-1 leu2*Δ *met15*Δ *ura3*Δ *ydj1*Δ *[pYX142-ydj1-DNAJA3 LEU2]*	*ydj1* deletion strain complemented with human DNAJA3 gene
*ydj1-*DNAJA4	*MATa his3-1 leu2*Δ *met15*Δ *ura3*Δ *ydj1*Δ *[pYX142-ydj1-DNAJA4 LEU2]*	*ydj1* deletion strain complemented with human DNAJA4 gene
*rad52*	*MATa his3-1 leu2*Δ *met15*Δ *ura3*Δ *rad52*Δ *[pYX142 LEU2]*	*rad52* deletion strain
WT + DNAJA3	*MATa his3-1 leu2*Δ *met15*Δ *ura3*Δ *ydj1*Δ *[pYX142- DNAJA3 LEU2]*	Wild type strain expressing the human DNAJA3 gene
WT + DNAJA4	*MATa his3-1 leu2*Δ *met15*Δ *ura3*Δ *ydj1*Δ *[pYX142- DNAJA4 LEU2]*	Wild type strain expressing the human DNAJA4 gene

**Table 2:** Growth Analysis of the HSP40s expressing strains.

Strain	Max growth (fold increase)	Growth_50_ (h)	Hill slope	Span
*ydj1*Δ	40.2	13.2	0.281	38.6
YDJ1	59.4	11.1	0.347	57.2
*ydj1-*DNAJA1	55.2	11.5	0.238	54.1
*ydj1-*DNAJA2	49.4	10.8	0.217	48.8
*ydj1-*DNAJA3	40.6	12.3	0.307	38.9
*ydj1-*DNAJA4	44.0	15.0	0.102	40.8

**Table 3:** Sensitivity of the HSP40s expressing strains to chemotherapeutic agents and heat shock.

Strain	Doxorubicin		Cisplatin		Heat shock	
	Survival (% ± SEM)	Sensitivity (fold)	Survival (% ± SEM)	Sensitivity (fold)	Survival (% ± SEM)	Sensitivity (fold)
*ydj1*Δ	6.4 ± 3.8	10	3.7 ± 2.0	7.3	0 ± 0	—
YDJ1	63.0 ± 7.8	1	27.0 ± 3.0	1.0	89 ± 26	1
DNAJA1	66.0 ± 5.0	1	38.0 ± 3.8	0.7	119 ± 5	0.7
DNAJA2	85.0 ± 8.0	0.7	72.0 ± 5.0	0.4	201 ± 24	0.4
DNAJA3	4.0 ± 3.0	15.8	1.0 ± 1.0	27.0	5 ± 5	18
DNAJA4	1.7 ± 2.0	37	4.0 ± 2.0	6.8	0 ± 0	—

**Table 4:** Sensitivity of the HSP40s expressing strains to etoposide and menadione.

Strain	Etoposide		Menadione			
			−NAc		+NAc	
	Survival (% ± SEM)	Sensitivity (fold)	Survival (% ± SEM)	Sensitivity (fold)	Survival (% ± SEM)	Sensitivity (fold)
*ydj1*Δ	104 ± 7.7	1	10 ± 2.5	3.7	21 ± 5.7	2.0
YDJ1	139 ± 6.4	1	37 ± 14.5	1	41 ± 16	1
DNAJA1	135 ± 3.3	1	47 ± 5.0	0.8	55 ± 7.0	0.8
DNAJA2	184 ± 8.0	0.7	109 ± 23.0	0.3	131 ± 29	0.3
*rad52*Δ	5 ± 4.8	29				

**Table 5:** Effect of DNAJA3 and DNAJA4 expression in a wild type background.

Strain	Doxorubicin		Heat shock	
	Survival (% ± SEM)	Sensitivity (fold)	Survival (% ± SEM)	Sensitivity (fold)
WT	63.3 ± 18.6	1	104 ± 4	1
WT-DNAJA3	20 ± 2.9	3.2	0 ± 0	—
WT-DNAJA4	4 ± 1.8	15.8	0 ± 0	—

## Data Availability

Data will be available by contacting the corresponding author. All strains and reagents used in the studies are available upon request.
